# An *Aedes aegypti*-Derived Ago2 Knockout Cell Line to Investigate Arbovirus Infections

**DOI:** 10.3390/v13061066

**Published:** 2021-06-03

**Authors:** Christina Scherer, Jack Knowles, Vattipally B. Sreenu, Anthony C. Fredericks, Janina Fuss, Kevin Maringer, Ana Fernandez-Sesma, Andres Merits, Margus Varjak, Alain Kohl, Esther Schnettler

**Affiliations:** 1 Bernhard Nocht Institute for Tropical Medicine, 20359 Hamburg, Germany; christina.scherer@bnitm.de; 2 German Centre for infection Research (DZIF), Partner Site Hamburg-Luebeck-Borstel-Riems, 20359 Hamburg, Germany; 3 MRC-University of Glasgow Centre for Virus Research, Glasgow G61 1QH, UK; JackKnowles01@outlook.com (J.K.); Sreenu.Vattipally@glasgow.ac.uk (V.B.S.); alain.kohl@glasgow.ac.uk (A.K.); 4 Icahn School of Medicine at Mount Sinai, New York, NY 10029, USA; anth.fredericks@gmail.com (A.C.F.); Kevin.Maringer@pirbright.ac.uk (K.M.); ana.sesma@mssm.edu (A.F.-S.); 5 Institute of Clinical Molecular Biology (IKMB), Kiel University, 24105 Kiel, Germany; j.fuss@ikmb.uni-kiel.de; 6 The Pirbright Institute, Pirbright GU24 0NF, UK; 7 Institute of Technology, University of Tartu, 50090 Tartu, Estonia; andres.merits@ut.ee (A.M.); margus.varjak@ut.ee (M.V.); 8 Faculty of Mathematics, Informatics and Natural Sciences, Universität Hamburg, 20148 Hamburg, Germany

**Keywords:** RNAi, exo-siRNA pathway, Ago2, Dcr2, knockout cell line, arbovirus replication

## Abstract

Mosquitoes are known as important vectors of many arthropod-borne (arbo)viruses causing disease in humans. These include dengue (DENV) and Zika (ZIKV) viruses. The exogenous small interfering (si)RNA (exo-siRNA) pathway is believed to be the main antiviral defense in arthropods, including mosquitoes. During infection, double-stranded RNAs that form during viral replication and infection are cleaved by the enzyme Dicer 2 (Dcr2) into virus-specific 21 nt vsiRNAs, which are subsequently loaded into Argonaute 2 (Ago2). Ago2 then targets and subsequently cleaves complementary RNA sequences, resulting in degradation of the target viral RNA. Although various studies using silencing approaches have supported the antiviral activity of the exo-siRNA pathway in mosquitoes, and despite strong similarities between the siRNA pathway in the *Drosophila melanogaster* model and mosquitoes, important questions remain unanswered. The antiviral activity of Ago2 against different arboviruses has been previously demonstrated. However, silencing of Ago2 had no effect on ZIKV replication, whereas Dcr2 knockout enhanced its replication. These findings raise the question as to the role of Ago2 and Dcr2 in the control of arboviruses from different viral families in mosquitoes. Using a newly established Ago2 knockout cell line, alongside the previously reported Dcr2 knockout cell line, we investigated the impact these proteins have on the modulation of different arboviral infections. Infection of Ago2 knockout cell line with alpha- and bunyaviruses resulted in an increase of viral replication, but not in the case of ZIKV. Analysis of small RNA sequencing data in the Ago2 knockout cells revealed a lack of methylated siRNAs from different sources, such as acute and persistently infecting viruses-, TE- and transcriptome-derived RNAs. The results confirmed the importance of the exo-siRNA pathway in the defense against arboviruses, but highlights variability in its response to different viruses and the impact the siRNA pathway proteins have in controlling viral replication. Moreover, this established Ago2 knockout cell line can be used for functional Ago2 studies, as well as research on the interplay between the RNAi pathways.

## 1. Introduction

Arboviruses are transmitted by biting arthropods such as ticks, midges, sandflies and mosquitoes. Mosquitoes transmit many medically important arboviruses, belonging to different virus families, including Zika virus (ZIKV, *Flaviviridae*), chikungunya virus (CHIKV, *Togaviridae*) and Rift Valley fever virus (RVFV, *Bunyavirales*, *Phenuiviridae*), to name a few. Upon infection, antiviral responses are triggered in mosquitoes to control viral replication [1]. The RNA interference (RNAi) mechanism is suggested to be the main antiviral defense against arboviruses in insects [2,3,4,5,6]. In mosquitoes, two RNAi pathways are triggered by viral infection resulting in the production of different virus-specific small RNAs: the exogenous 21 nt long small interfering (vsi)RNAs and the 24–30 nt long Piwi-interacting (vpi)RNAs. Typically, (v)piRNAs are produced by the so-called ping-pong production pathway, involving PIWI proteins and Argonaute 3, which results in the characteristic composition of vpiRNAs: U_1_ (anti-sense), A_10_ (sense) bias and 10 nt overlap of the sense and antisense piRNAs. The exogenous siRNA (exo-siRNA) pathway has been proposed to be the main host response to control virus infections in *Aedes aegypti* mosquitoes and derived cell lines [7]. This pathway is induced by viral replication-generated double-stranded RNA (dsRNA), which activates the response and involves the critical exo-siRNA effector proteins Dicer 2 (Dcr2) and Argonaute 2 (Ago2). These virus-derived dsRNAs are then cleaved into predominantly 21 nt vsiRNAs by Dcr2 and are subsequently loaded into Ago2 as part of the RNA-induced silencing complex (RISC). To the best of our current knowledge—based mostly on the model organism *Drosophila melanogaster*—siRNAs bound by Ago2 are methylated at their 3′ end and one strand of the siRNA duplex is degraded, while the remaining ‘guide strand’ is used to target and in turn cleave complementary viral RNA, resulting in reduced or impaired viral replication and translation.

Most arboviruses tested have been shown to produce vpiRNAs and vsiRNAs upon infection of mosquitoes or mosquito cell lines; however for some viruses, non-typical vpiRNAs have been reported [8]. In contrast, production of vsiRNAs has been observed upon infection with various arboviruses, supporting the general antiviral activity of the siRNA pathway [9,10,11,12].

In addition to arboviruses, insect-specific viruses (ISVs) that persistently infect mosquitoes or mosquito-derived cells have been reported in the past. Similar to arboviruses, persistent ISV infections have been shown to produce ISV-specific siRNAs and piRNAs in mosquitoes and derived cell lines. vsiRNAs have been reported for all investigated ISVs from different families, but vpiRNAs could only be found for some. For example, *Ae. aegypti*-derived Aag2 cells are known to be persistently infected with cell fusing agent virus (CFAV, *Flaviviridae*) and Phasi Charoen-like phasivirus (PCLV, *Phenuiviridae*). Both viruses produce vsiRNAs, but only PCLV showed typical vpiRNA production [13,14,15]. The biological function and possible antiviral activity of these ISV-specific vsiRNAs or vpiRNAs is currently unknown.

In contrast, key players of the siRNA response, Dcr2 and Ago2, are known to be antiviral against different arboviruses (*Togaviridae*, *Bunyavirales*, *Flaviviridae*) in *Ae. aegypti* [7]. Surprisingly, ZIKV seems to be an exception as silencing of Ago2 has no effect on ZIKV infection, suggesting that unlike Dcr2, Ago2 does not exert any antiviral activity [16,17,18]. Moreover, the magnitude of the observed increase during Ago2 knockdown for different viruses can vary, suggesting either differences in the importance of Ago2 antiviral activity between viruses [16,19,20,21,22], or effects of an incomplete knockdown and therefore residual activity of the target protein. Interestingly, Ago2 antiviral activity has always been linked to the siRNA pathway, with the incorporation of vsiRNAs used as guides to cleave complementary viral RNA. In contrast, Dcr2 could act independently of Ago2 solely through its dicing activity of the viral RNA or induction of other antiviral signaling pathways, like the Jak-STAT pathway through the expression of the Vago protein [23,24]. This highlights the considerable gaps that are still present regarding the antiviral RNAi response, and the contributions of each of these proteins.

To further elucidate these gaps, and to investigate the antiviral role of Dcr2 and Ago2 as main actors of the exo-siRNA pathway in *Ae. aegypti*, we used a newly engineered Ago2 knockout cell line to investigate the effects on arbovirus replication. Our findings validate the role of Ago2 as a key player in the antiviral response in *Ae. aegypti* during Semliki Forest virus (SFV; *Togaviridae*, *Alphavirus*) and Bunyamwera orthobunyavirus (BUNV; *Bunyavirales*, *Peribunyaviridae*), but not ZIKV, infection. These results confirm the importance of the exo-siRNA pathway in defending against arboviruses, but also raises new questions about its overall importance in the modulation of replication of different viruses in *Ae. aegypti*, or the potential for viral inhibition of parts of the pathway. This underlines the need to learn more about virus-vector interactions to support efforts for vector-based countermeasures against arboviruses.

## 2. Materials and Methods

### 2.1. Plasmids

Firefly luciferase (pIZ-Fluc) and *Renilla* luciferase (pAclE1-*Rluc*) expressing vectors have been previously described [25]. pPUb-myc-Ago2 (expressing *Ae. aegypti* Ago2) and pPUb-myc-eGFP have already been described elsewhere [19,26].

### 2.2. dsRNA Production

dsRNA targeting Firefly luciferase (FFluc), *Renilla* luciferase (*Rluc*), lacZ or eGFP were produced with the Megascript RNAi kit (Thermo Fisher Scientific Inc., Waltham, MA, USA) as previously described [19] (for primers, see Supplementary Table S1). In short, sequence-specific PCR products flanked with T7 RNA polymerase promoter sequences were produced and used for an in vitro transcription with T7 RNA polymerase. Following RNAse A and DNase I treatment, the obtained dsRNA was column-purified (Thermo Fisher Scientific Inc., Waltham, MA, USA).

### 2.3. Cells

Aag2-AF5 cells (ECACC 19022601) are a single cell clone of the *Ae. aegypti*-derived Aag2 cells [14] and Aag2-AF319 (ECACC 19022602) is a Dcr2 knockout derived from Aag2-AF5 cells, which have been already described [14,19]. Aag2-AF525 is an Ago2 knockout derived from Aag2-AF5 cells by CRISPR/Cas9.

All mosquito-derived cell lines used were grown in Leibovitz’s L-15 medium supplemented with 10% fetal calf serum (FCS), penicillin-streptomycin (P/S, final concentration 100 units/mL, 100 μg/mL, respectively) and 10% Tryptose Phosphate broth (TPB) at 28 °C. For RNAi reporter assays re-introducing Ago2, cells were grown in poly-l-lysine-coated flasks and experiments were performed without the addition of antibiotics.

Baby hamster kidney cells-21 (BHK-21) were maintained in Glasgow modified Eagle’s medium (GMEM) supplemented with 5% FCS, 1% P/S and 10% TPB. A549/BVDV-Npro cells, stably expressing the bovine viral diarrhea virus Npro protein (provided by R.E. Randall, University of St. Andrews, UK) [27] and Vero (ATCC CCL-81, *Cercopithecus aethiops*) cells were maintained in Dulbecco’s modified Eagle’s medium (DMEM) supplemented with 10% or 5% FCS, respectively, and 1% P/S. All mammalian cells (BHK21, A549/BVDV-Npro, Vero) cells were kept at 37 °C and an atmosphere with 5% CO_2_.

### 2.4. Viruses

BUNV-Nluc (expressing NanoLuc luciferase, Nluc) and the Brazilian ZIKV strain PE243 have been already described elsewhere [22,28]. Briefly, to produce BUNV-Nluc, the plasmids pTVT7RBUNM-NL, pT7riboBUNL(+), and pT7riboBUNS(+), encoding the BUNV anti-genome, were used. In pTVT7RBUNM-NL, a part of the BUNV NSm cytoplasmic domain was replaced by the Nluc sequence, resulting in chimeric NSm-Nluc fusion protein [22].

The plasmid pCMV-SFV6-2SG-Nluc containing reporter virus cDNA based on SFV strain 6 [29] was used for SFV6-2SG-Nluc reporter virus production. The reporter virus contains a duplicated sub-genomic promoter (spanning from position −37 to +17 in respect of sub-genomic RNA start site) placed immediately downstream of the structural reading frame of the SFV genome and followed by the sequence encoding for Nluc reporter. The plasmid pCMV-SFV4 [30] was used for the production of SFV4. Rescue of SFV4 from cDNA and titration have been previously described [19,30]. SFV4, SFV6-2SG-Nluc and BUNV-Nluc stocks were grown on BHK-21 cells, while ZIKV stocks were grown on A549/BVDV-Npro cells. Virus-containing supernatant was harvested after onset of a visible CPE, cleared by centrifugation and stored at −80 °C. Viral titers for SFV4, SFV6-2SG-Nluc and BUNV-Nluc were determined by plaque assays on BHK-21 cells using Avicel (0.6%)/MEM overlay with 2% FCS. ZIKV viral titers were determined by TCID50 on Vero cells.

### 2.5. Virus Detection via RT-qPCR

Cells grown in three wells of a 24-well plate were pooled for RNA isolation with Trizol according to manufacturer’s protocol. One-step quantitative RT-PCR for ZIKV and the ribosomal housekeeping gene S7 RNA was performed using specific primers (Supplementary Table S2), SYBR Green master mix (QuantiTect SYBR Green RT-PCR Kit, Qiagen, Germany) and a LightCycler 480 (Roche, Basel, Switzerland) according to manufacturer’s instructions. Results were analyzed using the ∆∆C_T_ method normalized to S7 housekeeping gene.

One-step quantitative RT-PCR for SFV was used for relative quantification with efficiency correction using the LightCycler 480 software (version 1.5.1.62; Roche, Basel, Switzerland), specific primers (Supplementary Table S2), SYBR Green master mix (QuantiTect SYBR Green RT-PCR Kit, Qiagen, Germany) and a LightCycler 480 (Roche, Basel, Switzerland) according to manufacturer’s instructions. Standard curves were created using serial dilutions of purified PCR products (SFV and S7 as reference).

### 2.6. Luciferase Assays

Relative luciferase activity was determined by using Dual-Luciferase Reporter Assay System, Firefly Luciferase Assay System and Nano-Glo Luciferase Assay System (Promega Corp., Fitchburg, WI, USA) in a GloMax luminometer following cell lysis in Passive Lysis Buffer (Promega Corp., Fitchburg, WI, USA) according to manufacturer’s protocols.

### 2.7. Production of the Aag2-Ago2 Knockout Cell Line Aag2-AF525

To create an Ago2 knockout cell line in *Ae. aegypti*-derived Aag2-AF5 cells (Aag2-AF525), the same set up as previously described for the production of Dcr2 knockout cells was used [19]. In short, a gRNA (AGAATGGCCTGGCGCCCAACAGG) targeting exon 2 of the Ago2 gene was cloned into the *Drosophila* CRISPR vector pAc-sgRNA-Cas9 [31], a kind gift from Ji-Long Liu (Addgene plasmid 49330). Aag2-AF5 cells were transfected with the corresponding plasmids, followed by selection, single-cell sorting and cell expansion. The final clone selected and used for these studies was designated Aag2-AF525.

### 2.8. RNAi Reporter Assays

To assess the Ago2 knockout efficiency, AF525 and AF5 cells were seeded in a 24-well plate (1.5 × 10^5^ cells/well). After 24 h, cells were transfected using 2 µL of DharmaFECT 2 (Horizon Discovery Ltd., Cambridge, UK) per well. Sixty nanograms of pIZ-Fluc and 10 ng pAcIE1-*Rluc* were co-transfected either with 1 ng siRNA (targeting FFluc or non-specific hygromycin B resistance gene as control [19]) or 10 ng dsRNA (targeting *Rluc* or eGFP (control)). For Ago2 reconstitution assays, AF5 and AF525 cells at a density of 2.4 × 10^5^ cells per well in poly-l-lysine-coated wells were transfected 24 h after seeding using 2 µL of DharmaFECT 2 (Horizon Discovery Ltd., Cambridge, UK) per well. Cells received 100 ng of pIZ-Fluc and 100 ng pAcIE1-*Rluc*, which were co-transfected with 10 ng dsRNA (targeting FFluc or non-specific lacZ). Additionally, cells were co-transfected with 500 ng per well plasmids expressing either myc-Ago2 or myc-eGFP (control). At 48 h post transfection (hpt), cells were lysed, and relative luciferase activity was measured using Dual-Luciferase Reporter Assay System (Promega Corp., Fitchburg, WI, USA).

### 2.9. Viral Replication Assays in Mosquito Cells

AF5, AF319 and AF525 were seeded at a density of 1.5 × 10^5^ cells/well in 24-well plates (SFV6-2SG-Nluc and BUNV-Nluc) or 3 × 10^5^ cells/well (ZIKV). Cells were infected 24 h post seeding at multiplicity of infection (MOI) of 1 (SFV6-2SG-Nluc and BUNV-Nluc) or MOI 0.1 (ZIKV) using 100 µL of virus dilution and were left to incubate for 1 h before additional L-15-supplemented medium was added (1 mL). At 48 h post infection (hpi), SFV- and BUNV-infected cells were lysed using Passive Lysis Buffer and luciferase activity was measured by using a Nano-Glo Luciferase Assay System (Promega Corp., Fitchburg, WI, USA). For ZIKV, total RNA from cells was isolated at 72 hpi by Trizol. ZIKV and ribosomal S7 RNA levels were determined by one-step quantitative RT-PCR (Qiagen, Germany).

### 2.10. Re-Introduction of Ago2 into Infected Cells

AF525 cells were seeded in a 96 well plate (5 × 10^4^ cells/well) and transfected 24 h later, with 500 ng of plasmid expressing myc-Ago2 or myc-eGFP (control), using 0.5 µL of DharmaFECT2 (Horizon Discovery Ltd., Cambridge, UK). Subsequently, 4 hpt cells were infected with SFV6-2SG-Nluc (MOI 0.5). Then, 48 hpi SFV-infected cells were lysed using Passive Lysis Buffer, and luciferase activity was measured by Nano-Glo Luciferase Assay System (Promega Corp., Fitchburg, WI, USA).

### 2.11. Statistical Analysis

Results are expressed as mean ± standard error of the mean (SEM). Statistical significance between groups was determined using Student’s *t*-test. Statistical analyses were carried out using GraphPad Prism 8.0 software (GraphPad, San Diego, CA, USA). *p*-values ≤ 0.05 were considered statistically significant.

### 2.12. β-Elimination Assay and Small RNA Sequencing

Methylation status of small RNAs was determined using β-elimination assay. AF5 and AF525 cells (1 × 10^6^ cells/well) were seeded in 6-well plates, followed by infection with SFV4 (MOI 10) and harvested 24 hpi. Total RNA was isolated with Trizol (Thermo Fisher Scientific Inc., Waltham, MA, USA) according to the manufacturer’s protocol, using glycogen as a carrier prior to isopropanol addition. Isolated total RNA samples were equally divided into two portions. Following this, 5 µL of 20× borate buffer and 12.5 µL of sodium periodate (or water in case of control sample instead of sodium periodate) were added and 100 µL RNase-free H_2_O. After 15 min, 10 µL of glycerol was added and incubated for a further 15 min at room temperature. Afterwards, samples were treated with 1 µL glycogen, 1/10 V of 3 M sodium acetate and 3× volume of 99.8% ethanol. Samples were transferred to a −20 °C freezer overnight for precipitation. Next day, samples were centrifuged for 15 min at 14,000 rpm at 4 °C. The supernatant was removed and again centrifuged for 5 min removing supernatant again afterwards. Subsequently, RNA was washed with 70% ethanol and dried pellets resuspended in borate buffer followed by 90 min incubation at 45 °C. RNA was purified with Monarch RNA Cleanup kit (50 µg) (New England Biolabs, Inc., Ipswich, MA, USA) according to the manufacturer’s protocol and sent for Illumina-based small RNA sequencing. For the first repeat, at least 1 µg of total RNA was sent for small RNA sequencing using an Illumina based system at BGI (BGI-tech solutions, Hongkong; BGISEQ-500) as previously described [32]. In short, total RNA was loaded on PAGE gel and RNA molecules of 18–35 nts were isolated, followed by adaptor ligation, RT-PCR with Super ScriptII to produce and enrich for cDNA fragments. PCR products were PAGE gel purified, followed by circularization. The single strand circle DNA was used as final library and, after validation on the bioanalyzer, DNA nanoballs were produced from the libraries with phi29 that in turn generated single end 50 base reads.

For the second repeat, 100 ng total RNA was used for library preparation with the NEXTFLEX^®^ Small RNA-Seq Kit v3 (PerkinElmer Inc., Waltham, MA, USA) according to manufacturer’s protocol. Here, instead of isolating RNAs of 18–35 nts, total RNA was used to generate DNA libraries, followed by purification of PCR products of the corresponding length by PAGE gel. Following library sequencing on one lane NovaSeq6000 SP v1.0 (2 × 50 bp) at IKMB (Kiel, Germany). Data from both runs was analyzed as previously described [33] by mapping small RNAs to virus genome/anti-genome: SFV4 (KP699763.1), CFAV (NC_001564.1), PCLV (KM001087.1, KM001086.1, KM001085.1). Besides, small RNAs were mapped to transposon elements using the TEfam transposon consensus sequence (https://tefam.biochem.vt.edu/tefam, accessed 30 May 2014) and the transcriptome of *Ae. aegypti* (*Ae*. *aegypti* Liverpool AGWG, version AaegL5.2 accessed 13 July 2020, https://vectorbase.org).

## 3. Results

Previous studies of RNAi key proteins Ago2 and Dcr2 in the context of arboviral infections were based on transient silencing approaches in mosquito-derived cell lines. Results obtained were often marked with considerable uncertainty about side effects caused by an incomplete knockdown of target proteins, especially for viruses where no antiviral activity for Ago2 was observed, like ZIKV. To counter this issue, a CRISPR/Cas9 gene knockout approach was pursued that has been successful in the past to create an *Ae. aegypti*-derived Dcr2 knockout cell line. The resulting Ago2 knockout cell line was called AF525.

### 3.1. Reporter-Based Silencing Assays in Knockout Cells

Following engineering and amplification of AF525 cells, their lack of a functional siRNA-based response was investigated using luciferase reporter assays, and treatment with either dsRNA or siRNA as a silencing inducer.

AF525 and AF5 (parental) cells were co-transfected with luciferase reporter plasmids expressing FFluc, or *Rluc* luciferase (internal control). For silencing induction, either dsRNA targeting FFluc or eGFP-specific (dsControl) or siRNAs targeting FFluc or hygromycin B-specific (siControl) were co-transfected. Reporter-based silencing was assessed by determining relative luciferase activity at 48 hpt.

**Figure 1:**
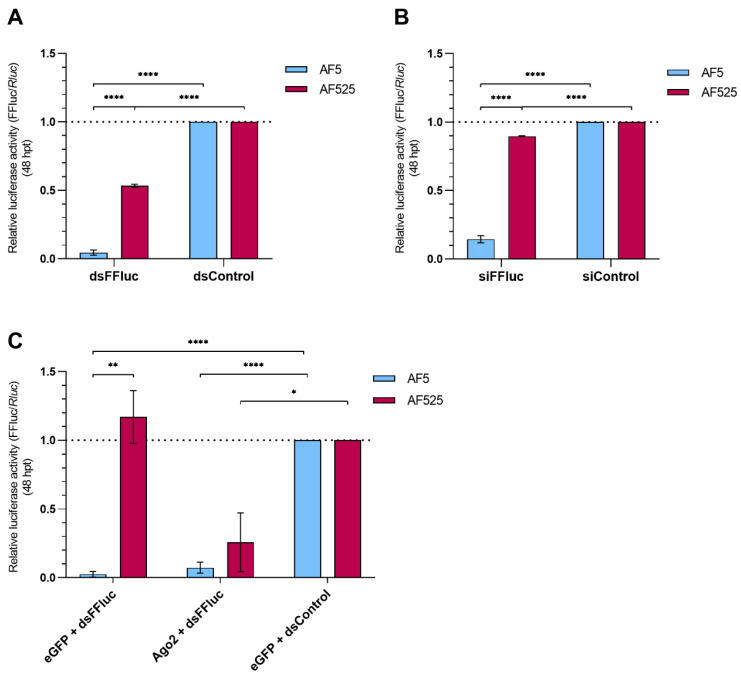
Characterization of AF525-Ago2 knockout cells by luciferase assay. Control cells AF5 and Ago2 knockout cells AF525 were co-transfected with FFluc and *Rluc* (internal transfection control) luciferase reporter plasmids together with either dsRNA (**A**) or siRNA (**B**) targeting FFluc or corresponding controls (dsRNA eGFP- or siRNA hygromycin B-specific; dsControl or siControl, respectively). (**C**) The experiment in panel A was repeated, but additionally, either Ago2 or eGFP (control) expression constructs were co-transfected and dsRNA against lacZ (control) was used as a control. At 48 hpt, relative luciferase activity (FFluc/*Rluc*) was determined and normalized to control cells. Means with SEM are shown for three independent experiments performed in triplicate (**** represents *p* ≤ 0.0001, ** represents *p* ≤ 0.01, * represents *p* ≤ 0.05, Student *t*-test).

As previously shown [19], luciferase activity was strongly reduced in AF5 cells transfected with FFluc-specific dsRNAs or siRNAs compared to control cells, indicating effective gene silencing (Figure 1A,B). Although, AF525 cells showed reduced luciferase activity in silenced cells compared to the controls, this was significantly less than was observed for the parental AF5 cell line, thus suggesting a reduced capacity to silence the target gene effectively (Figure 1A). In short, the reporter-based silencing assay suggests a consistent decrease in the silencing ability of AF525 cells, independent of the silencing inducer involved.

To ensure that the impaired silencing was due to the lack of Ago2, these experiments were repeated this time in the presence of Ago2 expressed from a plasmid expression system. AF5 or AF525 cells were co-transfected with expression constructs for Ago2 or eGFP (as control). Luciferase expression constructs (FFluc and *Rluc*) and dsRNA against the FFluc reporter or control lacZ were included as before, and luciferase activity was measured at 48 hpt. Again, luciferase activity was strongly reduced in AF5 cells transfected with dsRNA against the FFluc reporter compared to controls, independent of the presence of Ago2 expression constructs. In contrast, luciferase activity was only reduced in AF525 cells transfected with the Ago2 expression construct (Figure 1C). This reporter-based assay suggests that AF525 Ago2 knockout cells were unable to induce RNAi-based silencing through the exo-siRNA pathway, but that this functionality could be gained by re-introducing Ago2.

### 3.2. Small RNA Production in Ago2 Knockout Cells

Biogenesis of small RNAs in mosquito cells involves methylation to gain biological activity and stabilize small RNAs. In *D. melanogaster*, methylation is carried out in the RISC after dsRNA has been processed by Dcr2 and siRNAs are bound by Ago2. A methyl group (-CH_3_) is introduced onto the 2′ OH of the 3′ terminal nucleotide on each strand of the duplex by methyltransferase Hen1, creating an active RISC [34,35,36,37]. The methylation status of small RNAs can be determined by treating them with sodium periodate, which oxidates the 3′ terminus of those RNAs, and subsequent β-elimination [38]. RNAs with free 3′ OH are sensitive to further modification reactions through β-elimination reagents resulting in removal of the last nucleotide by cleavage of the terminal ribose [39]. This prevents the successful ligation of linkers to these small RNAs during the library preparation and therefore precludes their detection during small RNA sequencing analysis [39]. In contrast, methylated small RNAs are successfully ligated and sequenced. To further verify the loss of Ago2 expression in AF525 cells and the effect on siRNA methylation, β-elimination assays were performed on total RNA, followed by small RNA sequencing. Total RNA from AF525 or AF5 (as control) cells infected with SFV4 (MOI 10) were treated with sodium periodate, while control samples remained untreated. All samples were further treated with β-elimination reagents. Small RNAs from treated and untreated samples were then analyzed by Illumina-based Next Generation sequencing.

**Figure 2:**
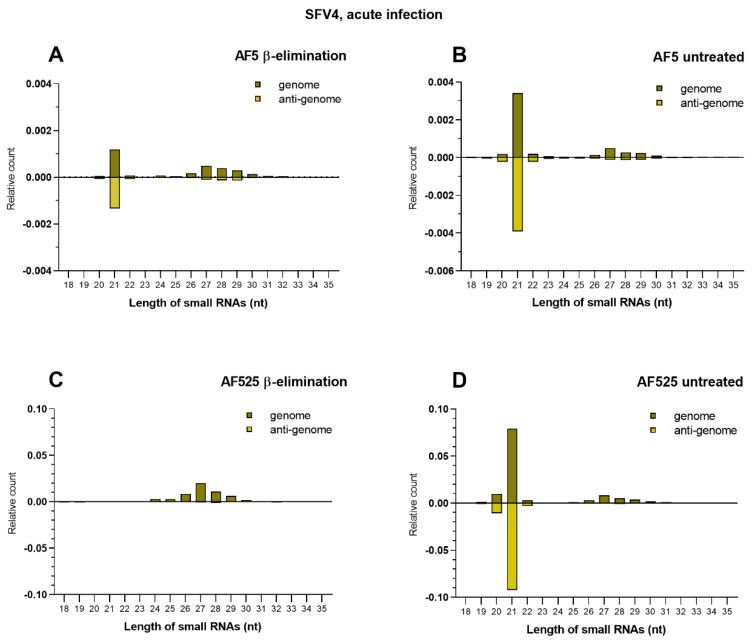
Length distribution of small RNAs in AF525 and AF5 cells treated with β-elimination reagents**.** NGS data of AF525 and AF5 cells was mapped to the SFV4 genome and anti-genome. Positive numbers are RNAs mapping to the sense strand of SFV4 (dark green), while negative numbers indicate RNAs mapping to the anti-sense strand of SFV4 (light green). *Y*-axis: relative count of small RNAs normalized to clean reads. (**A**) AF5 cells treated with complete β-elimination reagents. (**B**) AF5 β-elimination control. (**C**) AF525 cells treated with complete β-elimination protocol. (**D**) AF525 control. Two independent experiments were carried out and the results of one representative experiment is shown here (sequencing I).

Sequencing data showed that SFV4 infection resulted in the production of SFV4-specific 21 nt vsiRNAs and 24–30 nt vpiRNAs (Table 1, Figure 2) with the latter presenting the expected “ping-pong” production characteristics (A_10_/U_1_ bias and 10 nts overlap of antisense and sense small RNAs) in AF5 and AF525 untreated cells (Supplementary Figures S1 and S2). Similar to previous reports of AF5 cells [19], SFV4-specific vsiRNAs in AF525 and AF5 cells derived from the genomic and antigenomic RNA and map across the whole genome/anti-genome. However, in sodium periodate treated AF525 cells, there is a complete loss of SFV-specific siRNAs, which is not the case with AF5 cells. This indicates that the lack of Ago2 results in vsiRNAs being unmethylated and hence reacting with the reagent, subsequently preventing their detection via sequencing. In contrast, in AF5 cells, at least some siRNAs were protected from the chemical reaction and therefore could be detected.

SFV4-specific piRNAs in AF525 and AF5 cells, were mainly derived from the genomic RNA and mapped predominantly to the region where the sub-genomic promoter is located and at the 5′ end of the capsid coding region (Supplementary Figures S1 and S2). A discrepancy was observed for SFV4-specific piRNAs between the two replicates, produced by two different sequencing providers. In the second run (Table 1), the piRNA mapping and “ping-pong” amplification specific characteristics were not as obvious as in the first run, likely due to overall low piRNA read numbers. We believe that this is due to the differences in the workflow at the different sequencing facilities, such as the library kit used, library production and Next Generation sequencing equipment used by the two providers. This highlights the need for careful consideration of results received by small RNA sequencing analysis to ensure that certain characteristics not only arise due to technical bias.

**Figure 3:**
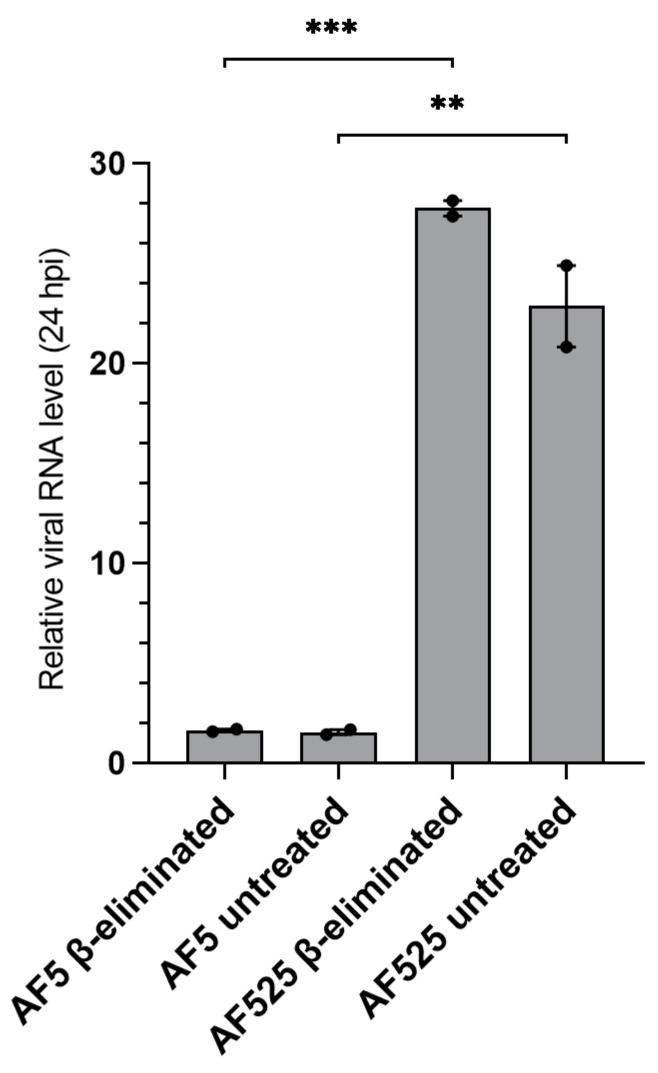
Relative viral RNA levels of SFV4-infected AF5 and AF525 cells treated with β-elimination reagents. Total RNA of SFV4-infected AF5 and AF525 cells was analyzed by RT-qPCR, using a standard curve, to determine viral RNA levels. Means with standard errors are shown for two independent experiments. Relative SFV4 amount was quantified using ribosomal S7 RNA as a housekeeper and normalized to untreated cells as control. *** indicates *p* ≤ 0.001; ** indicates *p* ≤ 0.01; Student *t*-test.

Intriguingly, a large increase in SFV4-specific vsiRNAs and vpiRNAs was detectable in untreated AF525 samples compared to AF5 cells (% 21 nts (SFV): 23.5–38.0-fold, % 28 nts (SFV): 5.0–15.3-fold, Table 1). This effect could be due to increased viral replication in AF525 cells, as the antiviral pathway is non-functional and can no longer limit SFV4 replication. Another reason might be the accumulation of small RNAs in the cell, as they are no longer processed by the RNAi response. To further examine those possibilities, the SFV4 genomic RNA was quantified. In AF525 cells, 28-fold increase in viral RNA was detected compared to AF5 cells; supporting the hypothesis that higher viral infection and replication was the main driver of the increased SFV4-specific small RNA production in AF525 cells (Figure 3).

To understand the involvement of Ago2 in the methylation of viral siRNAs in *Ae. aegypti*-derived cells, β-eliminated and untreated AF5 and AF525 cells were compared. miRNAs are known to be sensitive to β-elimination independent of Ago2 [40] and were therefore used as control to verify successful treatment. The number of miRNAs in β-eliminated samples (AF5 and AF525 cells) compared to untreated controls was strongly reduced, supporting a successful β-elimination treatment. Read numbers of the total 21 nt siRNAs (overall and SFV4-specific) in AF525 cells showed a strong decrease in β-eliminated samples (Table 1, Figure 2). In contrast, only a slight reduction was observed for AF5 β-eliminated cells, confirming the knockout of Ago2 in AF525 cells and the importance of Ago2 for small RNA methylation in *Ae. aegypti*-derived cells. The observed minor reduction in AF5 cells additionally suggested that not all 21 nt small RNAs are incorporated into RISC (Figure 2). Similar to overall siRNA levels, SFV4-specific vsiRNAs were strongly reduced in treated AF525 cells compared to untreated control samples. These findings confirmed that methylation of virus-derived siRNAs (from an acute virus infection) depends on Ago2 functionality (Table 1). In contrast, no reduction in SFV4-specific vpiRNAs was observed in β-eliminated samples (neither in AF5 nor AF525 cells). This confirms that piRNA methylation happens independently from Ago2 in *Ae. aegypti*, which renders piRNAs resistant to β-elimination [41], although it remains unclear why more 28 nt long piRNAs are methylated in treated samples compared to untreated controls (Table 1, Figure 2).

As Aag2 cells are known to be persistently infected with insect-specific CFAV and PCLV [13,14,15], it was expected that these viruses are also present in the AF525 cells. This was confirmed using the small RNA sequencing data in combination with the previously established virus discovery pipeline [32]. Mapping of reads to the CFAV genome revealed that vsiRNAs and a small quantity of vpiRNAs are produced equally in AF5 and AF525 untreated control cells which map to the viral genome and anti-genome (Table 2, Supplementary Figure S3). Results revealed that CFAV-derived vsiRNAs are also methylated as the fraction of 21 nt long small RNAs disappears in AF525 cells treated with β-elimination reagents compared to AF525 control (Table 2, Supplementary Figure S3). When comparing the untreated control cells, more CFAV-specific siRNAs were observed in AF525 than AF5 cells.

Results for PCLV show that vsiRNAs and vpiRNAs are also produced in AF5 and AF525 cells, mapping to the genome or anti-genome in a variable manner when comparing the first and second sequencing run (Table 2, Supplementary Figures S4–S6). It is noticeable that the amount of vpiRNAs was higher or almost equal to the amounts of vsiRNAs produced at least for the S- and M-segments of PCLV, especially in β-eliminated samples. Additionally, the amount of vpiRNAs appeared to increase in β-eliminated samples for all three segments. For the L-segment, a strong decrease in 21 nt vsiRNAs was observed in β-eliminated AF525, compared to untreated control samples. Similar to SFV4 and CFAV, more PCLV-specific vsiRNAs were detected in AF525 than AF5 cells.

**Figure 4:**
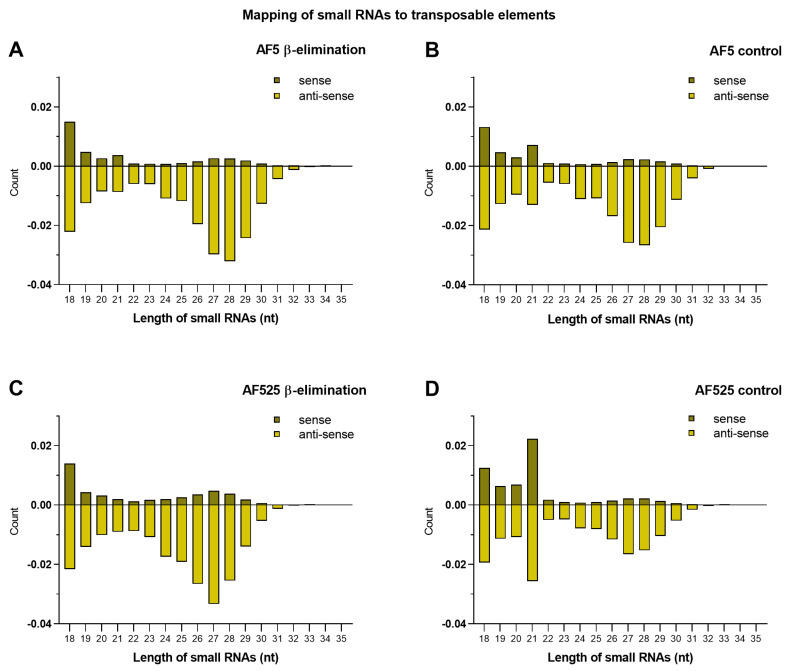
Length distribution of small RNAs from AF5 and AF525 cells mapping to TEs. Left panel side (**A**,**C**) shows treated samples of AF5 and AF525 cells, right panel side shows the results for untreated controls (**B**,**D**). *x*-axis displays the different lengths of small RNAs from 18 nt to 35 nt and *y*-axis displays the relative quantity of small RNAs normalized to clean reads. Positive numbers indicate RNAs mapping to the sense strand of TEs (dark green), while negative numbers indicate RNAs mapping to the anti-sense strand of TEs (light green). Two independent experiments were carried out, and the results of one representative experiment are shown here (run I).

The effect of Ago2 loss on the methylation of small RNAs originating from transposable elements (TEs) or the *Ae. aegypti* transcriptome was also analyzed. Sequencing analysis showed (Table 3, Figure 4) that siRNAs (21 nt) and piRNAs (24–30 nt) were produced from both the AF5 or AF525 genome and that a clear reduction of siRNAs mapping to TEs was observed in β-eliminated RNA from AF525 cells, hinting towards a dependency on Ago2 for TE-targeting 21 nt siRNA methylation. Noticeably, the amount of piRNAs mapping to transposable elements in AF5 and AF525 cells increased in treated cells similar to the previously documented increase of SFV4-mapped vpiRNAs. The effect is even stronger in AF525 cells where a strong increase of piRNAs was detectable, compared to AF5 untreated controls (Table 1).

Overall, the data suggested that methylation of piRNAs targeting TEs, which are natural targets of piRNAs, was not heavily affected by the lack of Ago2, unlike the endogenous siRNAs targeting TEs due the lack of siRNA methylation.

**Figure 5:**
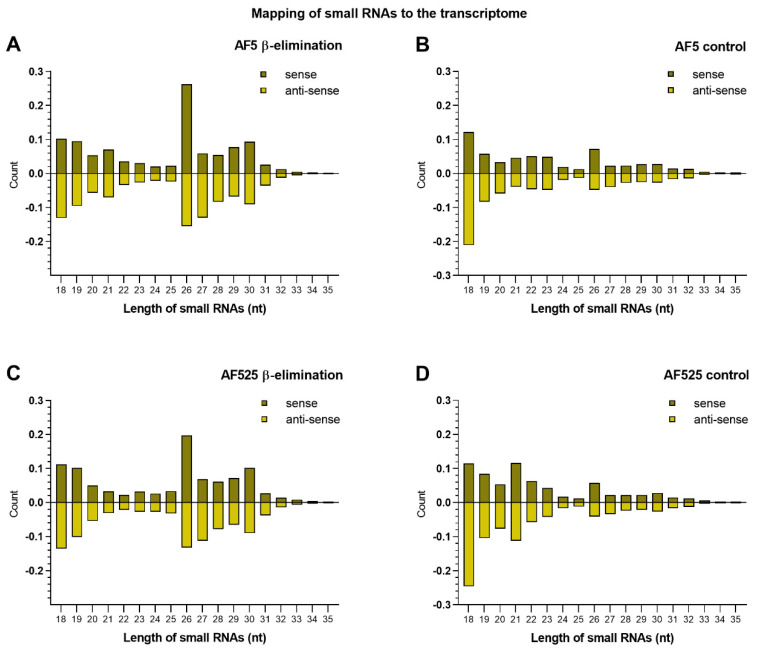
Length distribution of small RNAs from AF5 and AF525 cells mapping to the transcriptome of *Ae. aegypti* Liverpool AGWG, version AaegL5.2**.** Left panel side (**A**,**C**) shows treated samples of AF5 and AF525 cells, right panel side shows the results for untreated controls (**B**,**D**). *x*-axis displays the different lengths of small RNAs from 18 nt to 35 nt and *y*-axis displays the amount of small RNAs normalized to clean reads. Positive numbers are RNAs mapping to sense RNAs (dark green) while negative numbers map to anti-sense RNAs (light green). Two independent experiments were carried out and the results of one representative experiment are shown here (run I).

Mapping small RNAs of AF5 and AF525 cells to the transcriptome revealed that small RNAs with a very diverse length distribution were produced against both the sense and anti-sense transcriptome of AF5/AF525 cells (Figure 5). Overall, read counts of 21 nt siRNAs decreased in β-eliminated AF525 cells hinting towards a general effect of β-elimination treatment and the involvement of Ago2 in the methylation status of transcriptome-derived siRNAs (Figure 5C).

Taken together, analysis of small RNA sequencing data suggested that Ago2 is strongly involved in the methylation of not only virus-derived exogenous siRNAs, but also in methylating endogenous derived siRNAs targeting transposable elements or gene transcripts.

### 3.3. Effect of Ago2 on Viral Replication

In order to investigate the antiviral role of effectors in the exo-siRNA pathway (Dcr2, Ago2) in more detail, we determined viral replication levels for various arboviruses, including ZIKV (previously shown to be unaffected by Ago2 silencing [16]) in AF525 cells. Previously described Dcr2 knockout cells AF319 [19], AF525 and parental AF5 control cells were infected with luciferase-expressing SFV6-2SG-Nluc [19], BUNV-Nluc [22] (both MOI 1) and ZIKV (MOI 0.1). At 48 hpi, SFV6-2SG-Nluc and BUNV-Nluc infected cells were lysed, and relative luciferase activities were determined. Total RNA of ZIKV infected cells was isolated at 72 hpi and viral RNA copy number equivalents were determined by RT-qPCR.

**Figure 6:**
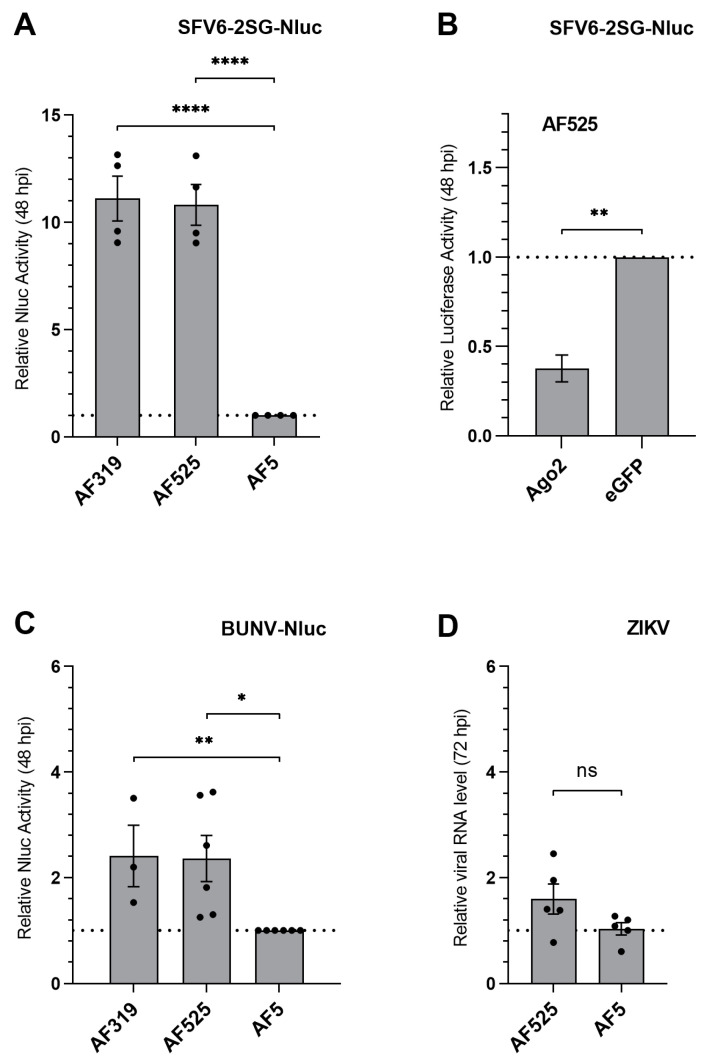
Viral replication in Ago2 and Dcr2 knockout cells, as well as in AF525 cells transfected with Ago2 expression plasmid. (**A**,**C**) AF525, AF319 and the parental cell line AF5 were infected with SFV6-2SG-Nluc (MOI = 1) or BUNV-Nluc (MOI = 1). After 48 h, cells were lysed to determine the relative luciferase activity and normalized to the AF5 control cells. The average with SEM from at least three independent experiments performed in triplicates are shown. (**B**) AF525 cells were transfected with myc-Ago2 or myc-eGFP (control) expression plasmids. At 4 hpt, cells were infected with SFV6-2SG-Nluc (MOI = 0.5), and 48 hpi cells were lysed to measure luciferase activity. The relative mean luciferase amounts of Nluc, normalized to eGFP transfected cells, with SEM, from three independent experiments conducted in triplicates are shown. (**D**) The mean relative ZIKV genomic RNA levels from five independent experiments in AF525 and AF5 cells infected with a MOI of 0.1 and total RNA isolation at 72 hpi is shown. Ribosomal S7 RNA was used as a housekeeper for the ∆∆C_T_ method with AF5 cells as a control. Dotted lines at 1 represent the controls used for normalization. **** indicates *p* < 0.0001, ** indicates *p* < 0.01, * indicates *p* < 0.05, ns indicates not significant, Student *t*-test.

Luciferase expression was significantly increased in AF525 and AF319 cells infected with SFV6-2SG-Nluc or BUNV-Nluc, compared to AF5 control cells (Figure 6B,C). No significant difference in viral replication levels was observed between Dcr2 and Ago2 knockout cells for SFV6-2SG-Nluc or BUNV-Nluc-infected cells, indicating that the absence of Dcr2 or Ago2 contributed to improved viral replication. For AF525 infected with ZIKV, cells accumulated 1.6-fold more viral RNA during the course of infection than AF5 control cells, although this was not statistically significant (Figure 6D). These data confirm our recent data [16] and suggest that Ago2 exerts only a minor effect, if any, in counteracting ZIKV replication.

Resubstituting Ago2 in the AF525 cell line through transfection of a myc-Ago2 expression construct prior to SFV6-2SG-Nluc infection (MOI 0.5), resulted in a decrease in luciferase compared to control cells (transfection of myc-eGFP construct, Figure 6B). These results confirm the increase in virus infection in AF525 cells is linked to the absence of Ago2 and thereby substantiates the antiviral activity of Ago2, at least against SFV infection.

## 4. Discussion

The exo-siRNA pathway is the main antiviral defense pathway in mosquitoes, with Dcr2 and Ago2 having major roles [19,21,42,43,44,45]. Silencing one of these key proteins during infection leads to an increase in viral replication for most arboviruses tested, including CHIKV, DENV and RVFV [7]. However, in some cases, an increase in virus replication was reported following Dcr2, but not Ago2, knockdown, in particular following infection with flaviviruses [16]. Whether this was due to residual Ago2 activity following incomplete transient silencing of Ago2, or a lack of antiviral activity by the exo-siRNA pathway was previously unclear.

To further assess the interplay of the exo-siRNA pathway and ZIKV replication, a CRISPR/Cas9 based gene knockout approach was pursued leading to an Ago2 knockout cell line, AF525. Reporter-based silencing assays showed that AF525 were not capable of gene silencing, regardless of the inducer molecule employed. Additionally, a β-elimination assay proved that methylation of endo- and exo-siRNAs performed by the Ago2-containing RISC complex was indeed defective.

Methylation is an important step during biogenesis of siRNAs. It establishes the biological activity and stability of RNAs, which originate from dicing of viral double-stranded transcripts or endogenous sources, such as structured transcripts or transposons by Dcr2 [35]. In *D. melanogaster*, siRNA methylation occurs in an Ago2-dependent manner [36]. Similarly, Ago2 knockout in *Ae. aegypti*-derived cells, revealed a broad effect on the methylation of viral small RNAs. In Ago2 knockout cells, vsiRNAs and endo-siRNAs were no longer methylated, in contrast to vpiRNAs, which were largely unaffected. These results indicate that methylation of vsiRNAs as well as endo-siRNAs in this *Ae. aegypti*-derived cell line is dependent on Ago2.

Our experiments underline the importance of RNAi and particularly the exo-siRNA pathway for the control of arboviral replication. Knocking out Ago2 or Dcr2 in *Ae. aegypti*-derived cell lines resulted in a strong enhancement of SFV replication, which is in accordance with other knockdown studies [9,19], indicating that both key players of the siRNA pathway are clearly required. Re-introducing Ago2 in the knockout cells led to a significant decrease compared to control cells, demonstrating that the positive effect on SFV replication was due to the lack of Ago2 in AF525 cells, as previously shown for Dcr2 in the AF319 [19].

Similar results were observed for BUNV-Nluc, which was positively affected in Dcr2 and Ago2 knockout cells, although the overall increase was less than that determined for SFV. Previous studies have shown that the quantity of vsiRNAs produced in BUNV-infected cells is less than that produced in SFV-infected cells [9,22]. It could be that BUNV evades the main antiviral response in mosquitoes either by hiding from its main actors or through active suppression of host responses. This remains to be investigated.

For many mosquito-borne flaviviruses, replication increases in *Ae. aegypti* or *D. melanogaster* in vitro as well as in vivo in the absence of an exo-siRNA response [11,21,44]. In addition, previous studies observed ZIKV-specific siRNAs in infected mosquito cells and increased ZIKV replication in a Dcr2 knockout cell line, which provided supporting evidence for the antiviral activity of the exo-siRNA pathway against ZIKV [16,46]. However, ZIKV replication was unchanged in previously described Ago2 knockdown [16], as well as knockout cells, suggesting that Ago2 lacks antiviral activity against ZIKV. The discrepancy of the two key siRNA pathway proteins and their ability to act antiviral against ZIKV remains to be further investigated. Possible explanations for the lack of Ago2 antiviral activity could be the inaccessibility of ZIKV RNA, the expression of an RNA silencing suppressor (VSR) by ZIKV or the compensation of Ago2 activity by another Argonaute protein. Besides, the differences in hierarchy in the exo-siRNA pathway and additional protein interaction of Dcr2 and Ago2 could also explain, at least partly, the differences seen for the different knockout cells upon virus infection.

Various studies indicate a combination of separated replication sites to protect viral RNA intermediates passively from host cell antiviral mechanisms and active protection by suppressor functions of viral proteins [47,48,49,50,51] as even protected replication sites need to be connected to the cytosol. The fact that ZIKV-specific siRNAs are produced in infected cells and mosquitoes prove that ZIKV dsRNA must be accessible for dicing by Dcr2. However, it is not known if the same is true for the accessibility of the active RISC. For several medically important flaviviruses, the capsid protein has been reported to be a VSR, including DENV, yellow fever virus and ZIKV [48]. However, other studies of ZIKV capsid protein showed that the VSR activity is independent from the exo-siRNA pathway [16] supporting the observed lack of Ago2 antiviral activity for ZIKV.

Differences in viral replication efficiency, quantity of viral specific small RNAs and VSR activities, including the number of VSRs encoded by a virus or the stage where the VSR acts in the RNAi pathway, could explain discrepancies of antiviral RNAi proteins for viruses even from the same virus family. For example, in contrast to ZIKV, knockdown of Ago2 led to an increase in DENV (also a member of the *Flavivirus* genus) [11,41]. However, the NS2A protein and capsid protein has been lately suggested to act as a VSR [52] supporting the finding that DENV2 is still susceptible to an antiviral RNAi response [11,53].

It is not known if another Argonaute protein, i.e., Ago1 of the miRNA pathway, in *Ae. aegypti* is able to compensate for Ago2 antiviral activity, although our results would suggest this is not the case. As knockdown and knockout of Ago2 normally results in increased virus replication, this would be specific for ZIKV infection.

Previously, it was reported that Dcr2 also acts antiviral by mediating a signaling pathway that results in the expression of Vago and activates the antiviral Jak-STAT pathway; thereby inhibiting WNV infection in *Culex*-derived cells [24]. Knockout of Dcr2 would therefore affect two separate antiviral pathways, in contrast to Ago2 knockout. However, the involvement of Vago through Dcr2 activity are an unlikely explanation for the observed results of ZIKV in Ago2 knockout versus Dcr2 knockout cells, as recent results have shown that virus infection is unable to induce Vago expression in *Ae. aegypti*-derived cells [54].

Similarly to SFV, more PCLV and CFAV specific small RNAs were detected in the Ago2 knockout cells compared to controls. This suggests that the antiviral activity of Ago2 is not only effective against acute infections, such as SFV, but also persistent infections. However, it is yet to be verified if an increase in titer for both PCLV and CFAV is observed within AF525 cells. Further experiments are needed to verify this. However, vsiRNAs against PCLV and CFAV showed an increase in treated AF5 cells, suggesting methylation and biological activity of the vast majority of them. In contrast, SFV4 specific vsiRNAs decreased, indicating that a lower percentage of them were methylated and thereby biologically active. Overall, a higher proportion of PCLV-specific vsiRNAs were detected in AF525 compared to AF5 cells. Whether this points towards Ago2-independent methylation of 21 nt vsiRNAs derived from the S- and M-segment, while those derived from the L-segment are methylated in an Ago2 dependent manner, remains elusive. It is currently unknown if the lower expression rate of the L-segment could have attributed to the production of less dsRNA substrate. This is yet to be investigated.

Taken together, our findings indicate that acute infection of SFV and BUNV replication is targeted and modulated by the exo-siRNA pathway, with contributions from both Dcr2 and Ago2. Small RNA sequencing data also suggests that the persistent PCLV and CFAV infections are targeted by the exo-siRNA pathway and effected by the antiviral activity of Ago2; however, follow up experiments are needed to prove this antiviral activity. It is becoming increasingly clear that arboviral families share similar characteristics with their interactions with mosquito antiviral responses, although there are also some strong differences that need to be explored further. The newly created Ago2 knockout cell line provides the opportunity to investigate functions within the exo-siRNA pathway further.

## 5. Conclusions

An Ago2-deficient *Ae. aegypti* cell line was produced by CRISPR/Cas9 and used to determine the importance of Ago2 in the antiviral siRNA response against different arboviruses, as well as the requirement for siRNAs to be methylated in order for silencing to occur. The loss of Ago2 resulted in a lack of methylated siRNA molecules of different origins, including those derived from exogenous virus, persistent viruses, TEs and genomic transcripts. SFV and BUNV replication both benefitted from the lack of exo-siRNA pathway proteins. In contrast, ZIKV replication showed no significant increase upon Ago2 knockout. Therefore, this cell line provides a useful tool to study the impact of Ago2 on specific viruses and could be used to investigate Ago2 functions further.

## Figures and Tables

**Table 1:** Analysis of clean reads for SFV4-infected AF5 and AF525 samples treated with β-elimination reagents. Total number of clean reads is listed for each sample, as well as percentage of miRNAs detected in the sample. Furthermore, the percentage of total reads of 21 nt (% 21 nt) and 28 nt long RNAs (% 28 nt) and the share of those RNAs mapping to SFV4, termed % 21 nt (SFV) and % 28 nt (SFV), respectively, are also shown. As a representative for siRNAs, 21 nt reads are shown, and 28 nt long reads as a representative for piRNAs. Data from two independent sequencing runs are listed.

Sample	Clean Reads	% miRNAs	% 21 nt	% 28 nt	% 21 nt (SFV)	% 28 nt (SFV)
AF5 β-eliminated I	28,154,509	0.13	2.56	20.97	0.25	0.05
AF5 β-eliminated II	22,173,319	1.89	0.59	1.62	0.68	0.01
AF5 untreated I	27,608,736	9.54	7.86	16.76	0.73	0.04
AF5 untreated II	47,416,333	17.11	0.50	1.09	0.36	0.01
AF525 β-eliminated I	27,801,658	0.03	1.6	18.08	0.10	1.20
AF525 β-eliminated II	27,191,855	1.27	0.86	2.36	0.14	0.14
AF525 untreated I	27,053,952	5.77	32.43	10.3	17.15	0.61
AF525 untreated II	40,043,576	12.41	0.43	1.01	13.79	0.05

**Table 2:** Analysis of clean reads in AF5 and AF525 cells persistently infected with CFAV and PCLV and treated with β-elimination reagents. Percentage of total reads of 21 nt (% 21 nt) and 28 nt (% 28nt) long RNAs mapping to CFAV or PCLV S-, M- and L-segment. Small RNAs of 21 nt are used as a representative for siRNAs and 28 nt long reads for piRNAs. Data from two independent sequencing runs are listed.

Sample	% 21 nt (CFAV)	% 28 nt (CFAV)	% 21 nt (PCLV-S)	% 28 nt (PCLV-S)	% 21 nt (PCLV-M)	% 28 nt (PCLV-M)	% 21 nt (PCLV-L)	% 28 nt (PCLV-L)
AF5 β-eliminated I	0.07	0.02	0.07	0.84	0.01	0.21	0.01	0.01
AF5 β-eliminated II	0.37	0.01	0.53	2.04	0.06	0.23	0.04	0.02
AF5 untreated I	0.17	0.01	0.05	0.56	0.02	0.14	0.01	0.01
AF5 untreated II	0.12	0.01	0.18	0.55	0.02	0.08	0.01	0.01
AF525 β-eliminated I	0.05	0.11	0.03	0.28	0.02	0.15	0.00	0.01
AF525 β-eliminated II	0.12	0.11	0.83	4.61	0.06	0.50	0.01	0.04
AF525 untreated I	1.34	0.05	0.11	0.16	0.05	0.07	0.05	0.01
AF525 untreated II	1.96	0.03	0.84	0.95	0.17	0.12	0.15	0.01

**Table 3:** Analysis of β-eliminated small RNA samples mapping to transposable elements of the AF5/AF525 genome and the *Ae. aegypti* transcriptome. Total number of clean reads is listed for each sample as well as the percentage of total reads of 21 nt (% 21 nt) and 28 nt long RNAs (% 28 nt) mapping to TEs and the share of those RNAs mapping to the transcriptome of the cell. Small RNAs of 21 nt are used as a representative for siRNAs and 28 nt long reads as a representative for piRNAs. Data from two independent sequencing runs are listed.

		TEs		Transcriptome	
Sample	Clean Reads	% 21 nt	% 28 nt	% 21 nt	% 28 nt
AF5 β-eliminated I	28,154,509	1.24	3.47	4.80	11.18
AF5 β-eliminated II	22,173,319	4.20	4.17	14.10	13.80
AF5 untreated I	27,608,736	2.01	2.90	9.19	10.65
AF5 untreated II	47,416,333	1.58	1.41	8.64	5.14
AF525 β-eliminated I	27,801,658	1.09	2.93	2.82	11.45
AF525 β-eliminated II	27,191,855	1.48	4.33	6.44	13.94
AF525 untreated I	27,053,952	4.80	1.74	15.44	8.08
AF525 untreated II	40,043,576	8.36	1.26	22.93	4.49

## Data Availability

The RNA sequencing data generated here is available in the NCBI Sequence Read Archive (PRJNA734154). The RT-qPCR and luciferase data is available under http://dx.doi.org/10.5525/gla.researchdata.1154, accessed on 19 May 2021.

## Supplementary Materials

The following are available online at https://www.mdpi.com/article/10.3390/v13061066/s1, Figure S1: Characteristics of β-eliminated small RNAs of AF5 cells (sequencing run I), Figure S2: Characteristics of β-eliminated small RNAs of AF525 cells (sequencing run I), Figure S3: Length distribution of small RNAs in AF5 and AF525 cells treated with β-elimination reagents mapping to CFAV (sequencing run I), Figure S4: Length distribution of small RNAs in AF5 and AF525 cells treated with β-elimination reagents mapping to the S-segment of PCLV (sequencing run I), Figure S5: Length distribution of small RNAs in AF5 and AF525 cells treated with β-elimination reagents mapping to the M-segment of PCLV (sequencing run I), Figure S6: Length distribution of small RNAs in AF5 and AF525 cells treated with β-elimination reagents mapping to the L-segment of PCLV (sequencing run I), Table S1: Primer sequences for dsRNA production, Table S2: Primer sequences for RT-qPCR.
