# Supplementary material for: An Aedes aegypti-Derived Ago2 Knockout Cell Line to Investigate Arbovirus Infections
Source: Viruses. 2021 Jun 3;13(6):1066. doi: 10.3390/v13061066 (PMC8227176; doi:10.3390/v13061066)
Supplement: Supplementary file 1 [file viruses-13-01066-s001.zip › viruses-1221421-supplementary.pdf]

Supplementary materials

Table S1. Primer sequences for dsRNA production. All sequences are shown 5′-3′. Underlined bases indicate the T7 RNA polymerase promoter sequence.

Target	Forward/reverse primer
dseGFP [53]	GTAATACGACTCACTATAGGGGGCGTGCAGTGCTTCAGCCGC<br>GTAATACGACTCACTATAGGGGTGGTTGTCGGGCAGCAGCAC
dslacZ [54]	TAATACGACTCACTATAGGGGTCGCCAGCGGCACCGCGCCTTTC<br>TAATACGACTCACTATAGGGCCGGTAGCCAGCGCGGATCATCGG
dsFFluc [53]	GTAATACGACTCACTATAGGGACTTACGCTGAGTACTTC<br>GTAATACGACTCACTATAGGGGAAATCCCTGGTAATCCG
dsRluc [55]	TAATACGACTCACTATAGGGATGACTTCGAAAGTTTATGATCCAG<br>TAATACGACTCACTATAGGGCTGCAAATTCTTCTGGT TCTAACTTTC

Table S2. Primer sequences for RT-qPCR [13]. All sequences are shown 5′-3′.

Target	Forward/reverse primer
ZIKV	GTTGTCGCTGCTGAAATGGA<br>GGGGACTCTGATTGGCTGTA
SFV	GCAAGAGGCAAACGAACAGA<br>GGGAAAAGATGAGCAAACCA
S7	CCAGGCTATCCTGGAGTTG<br>GACGTGCTTGCCGGAGAAC

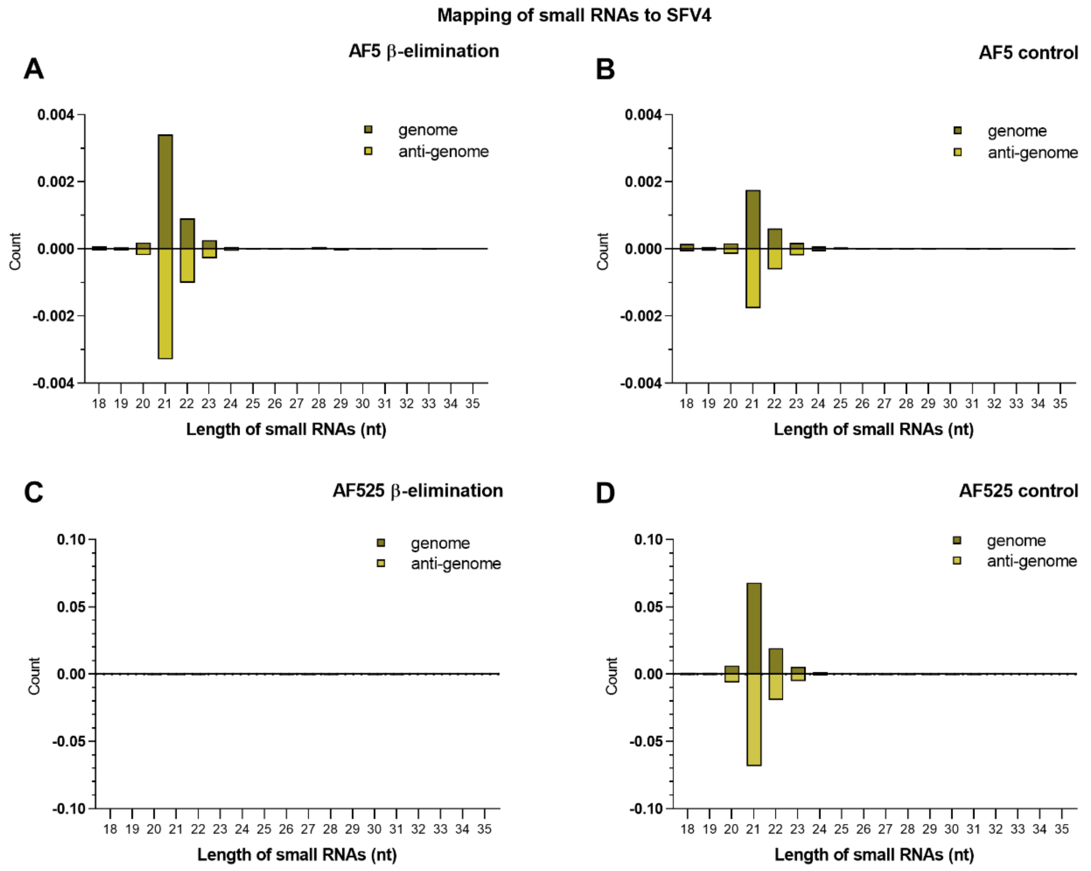

Figure S1. Length distribution of small RNAs in AF525 and AF5 cells treated with β-elimination reagents (sequencing run II). NGS data of AF525 and AF5 cells was mapped to the SFV4 genome and antigenome. Dark yellow bars indicate sequences mapping to the genome of SFV4 while light yellow bars map to the anti-genome of the virus. x-axis: length of

small RNAs, y-axis: relative count of small RNAs normalized to clean reads. A: AF5 cells treated with complete β-elimination reagents. B: AF5 β-elimination control. C: AF525 cells treated with complete β-elimination protocol. D: AF525 control. Two independent experiments were carried out and the results of one representative experiment are shown here.

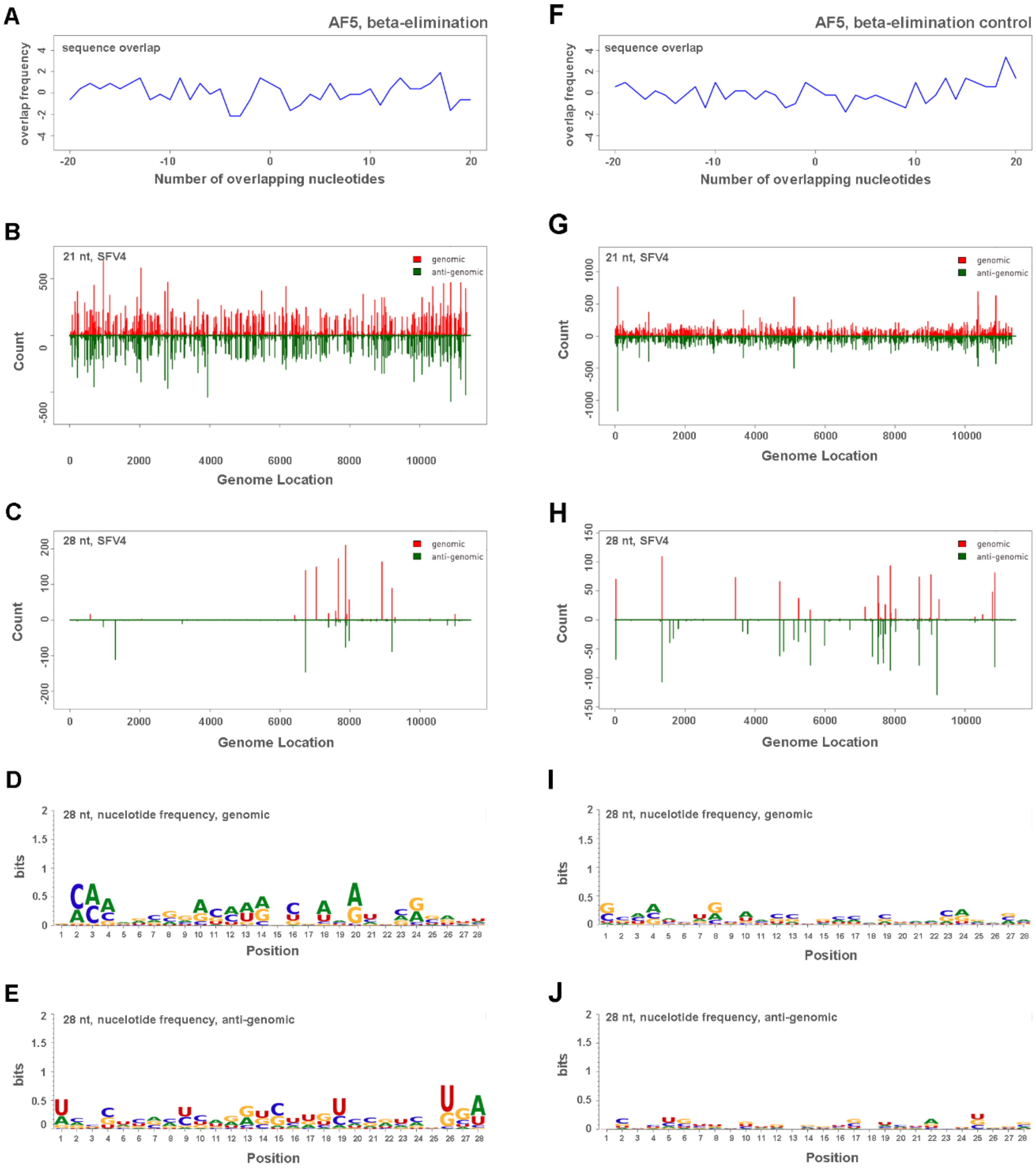

Figure S2. Characteristics of β-eliminated small RNAs of AF5 cells (sequencing run II). Small RNAs of AF5 cells treated with β elimination reagents (A-E) and control (F-J). A, F: sense and anti-sense sequence overlap of virus-derived piRNAs (25-29 nt). B, G: vsiRNA (21 nt) length distribution along the SFV4 genome (red, mapped to the genome; green, mapped to the antigenome). C, H: vpiRNA (28 nt) length distribution over the SFV4 genome (red, mapped to the genome; green, mapped to the antigenome). D, E, I, J: Relative nucleotide frequency and conservation per position of the 28 nt long piRNAs mapping to the SFV4 genome (D, I) or antigenome (E, J). Two independent experiments were carried out and the results of one representative experiment are shown here.

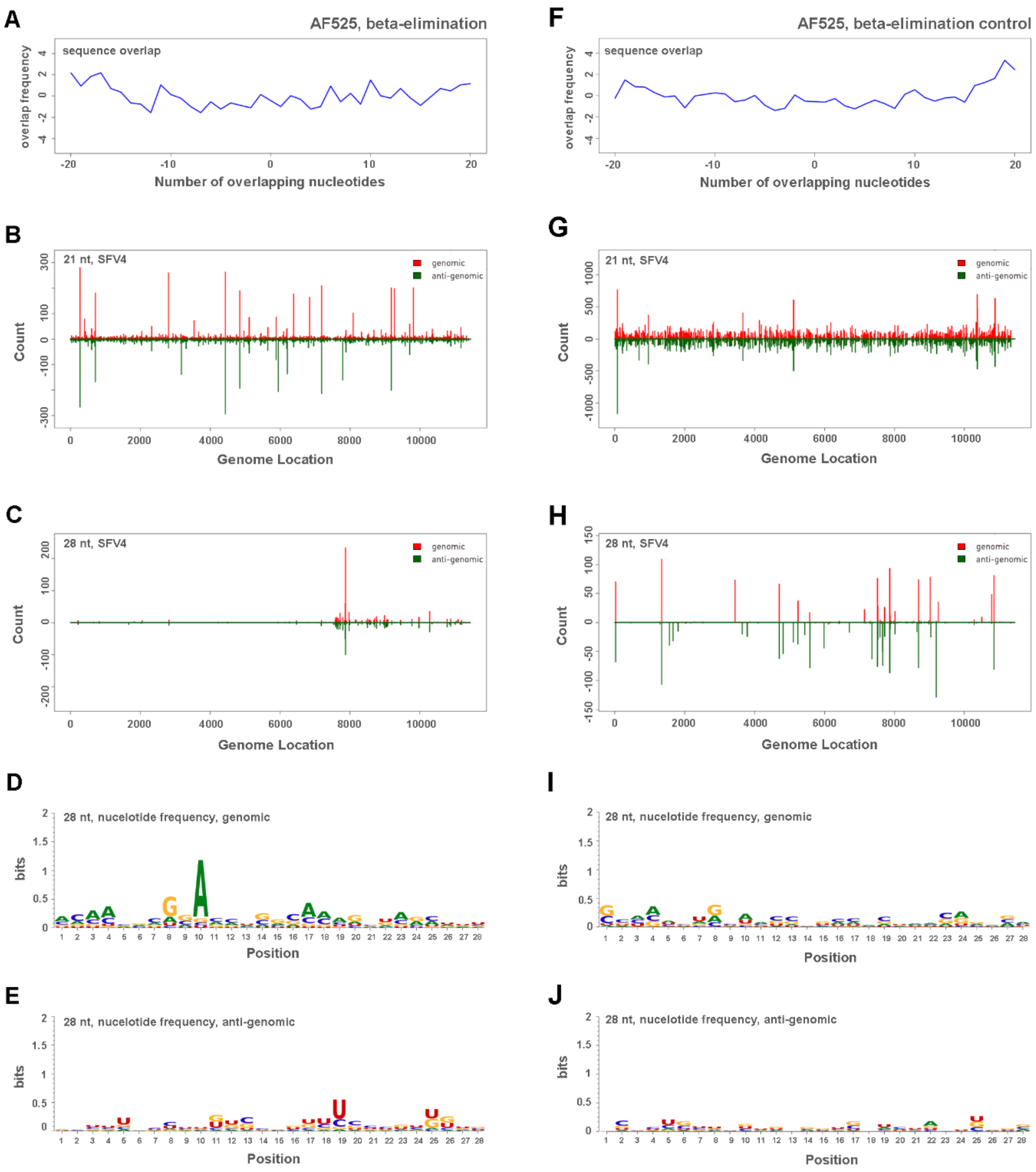

Figure S3. Characteristics of β-eliminated small RNAs of AF525 cells (sequencing run II). Small RNAs of AF525 cells treated with β elimination reagents (A-E) and control (F-J). A, F: sense and anti-sense sequence overlap of virus-derived piRNAs (25-29 nt). B, G: vsiRNA (21 nt) length distribution along the SFV4 genome (red, mapped to the genome; green, mapped to the antigenome). C, H: vpiRNA (28 nt) length distribution over the SFV4 genome (red, mapped to the genome; green, mapped to the antigenome). D, E, I, J: Relative nucleotide frequency and conservation per position of the 28 nt long piRNAs mapping to the SFV4 genome (D, I) or antigenome (E, J). Two independent experiments were carried out and the results of one representative experiment are shown here.

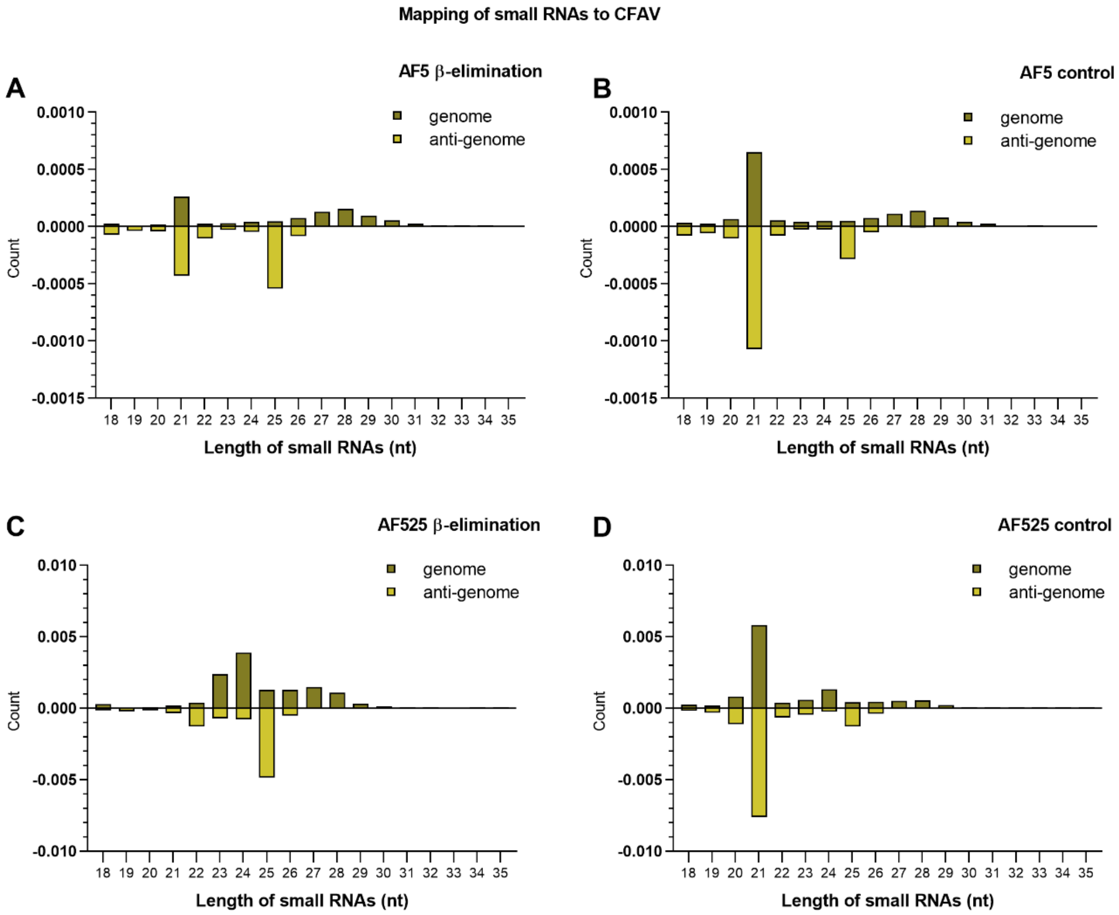

Figure S4. Length distribution of small RNAs in AF5 and AF525 cells treated with β-elimination reagents mapping to CFAV (sequencing run I). NGS data of AF525 and AF5 cells was also mapped against the genome of CFAV. Dark yellow bars indicate sequences mapping to the genome of CFAV while light yellow bars map to the anti-genome of the virus. x-axis: length of small RNAs, y-axis: relative count of small RNAs normalized to clean reads. A: AF5 cells treated with complete β-elimination reagents. B: AF5 β-elimination control. C: AF525 cells treated with complete β-elimination protocol. D: AF525 control.

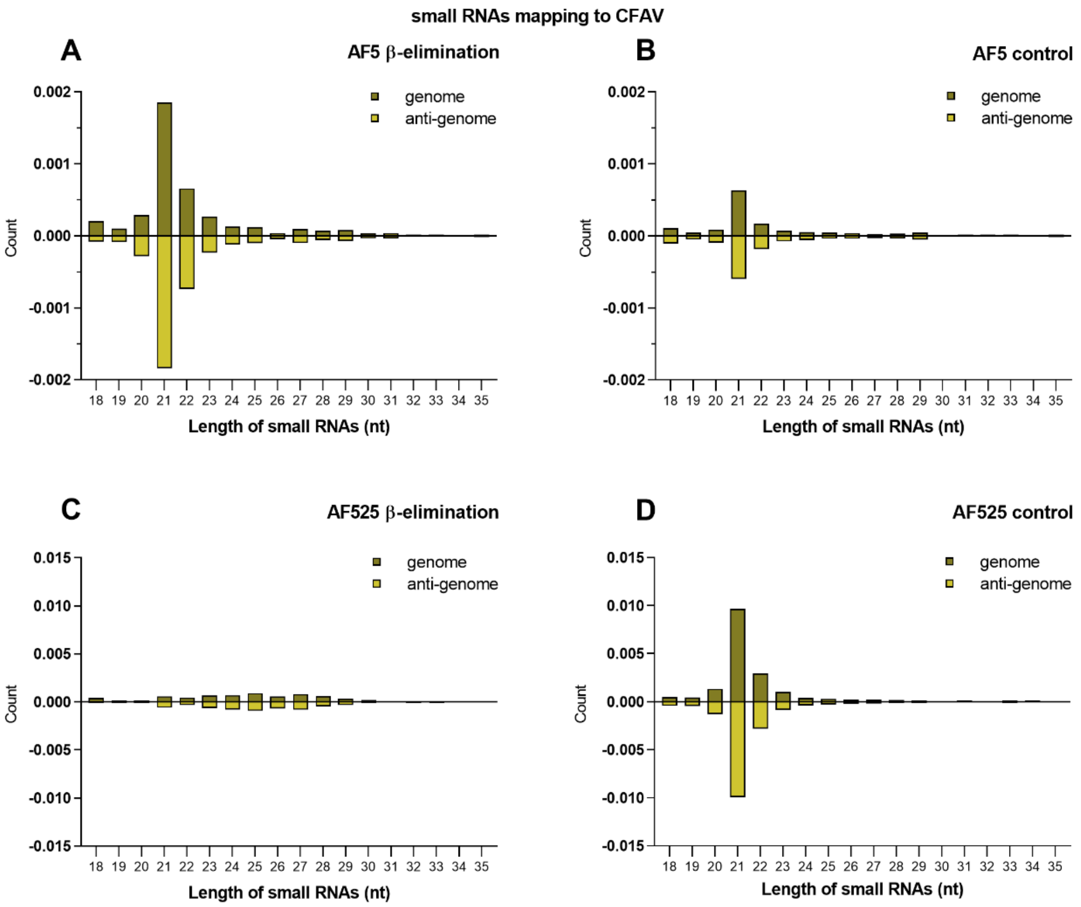

Figure S5. Length distribution of small RNAs in AF5 and AF525 cells treated with β-elimination reagents mapping to CFAV (sequencing run II). NGS data of AF525 and AF5 cells was also mapped against the genome of CFAV. Dark yellow bars indicate sequences mapping to the genome of CFAV while light yellow bars map to the anti-genome of the virus. x-axis: length of small RNAs, y-axis: relative count of small RNAs normalized to clean reads. A: AF5 cells treated with complete β-elimination reagents. B: AF5 β-elimination control. C: AF525 cells treated with complete β-elimination protocol. D: AF525 control.

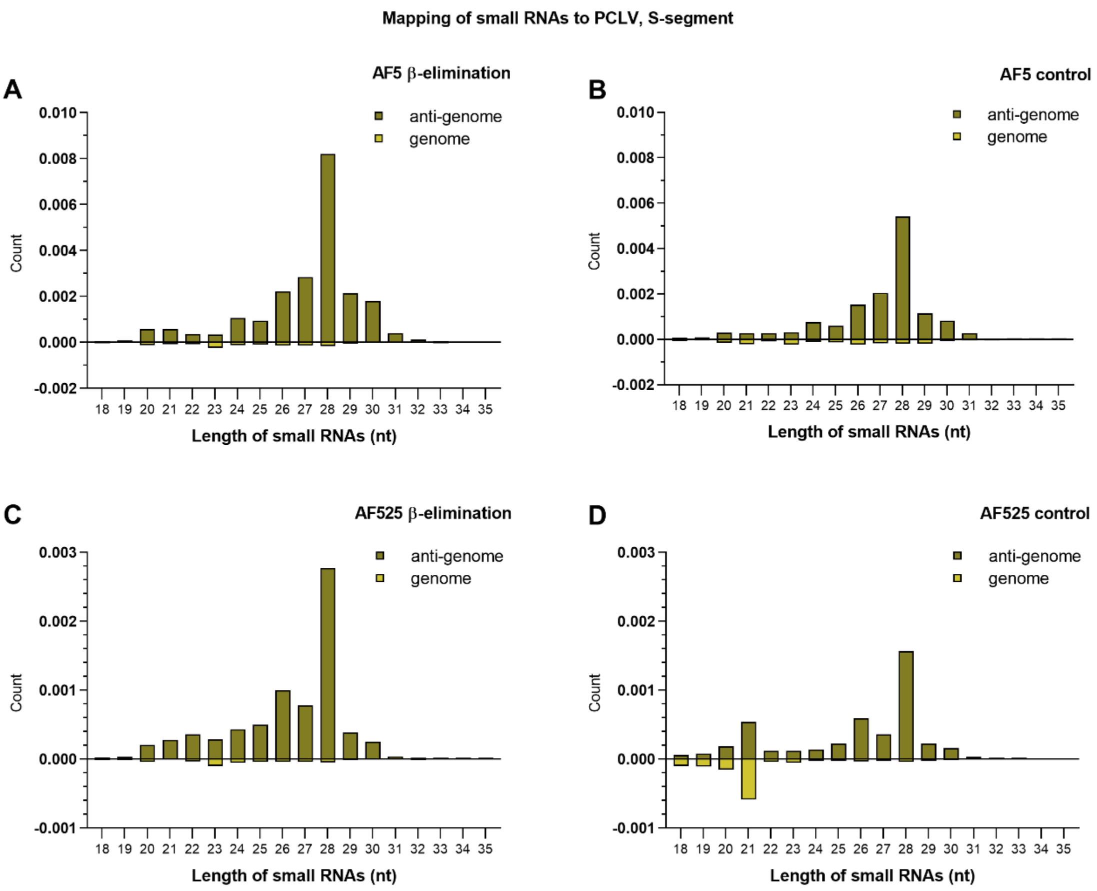

Figure S6. Length distribution of small RNAs in AF5 and AF525 cells treated with β-elimination reagents mapping to the S-segment of PCLV (sequencing run I). NGS data of AF525 and AF5 cells was also mapped against the genome of the PCLV S-segment. Dark yellow bars indicate sequences mapping to the genome of PCLV while light yellow bars map to the anti-genome of the virus. x-axis: length of small RNAs, y-axis: relative count of small RNAs normalized to clean reads. A: AF5 cells treated with complete β-elimination reagents. B: AF5 β-elimination control. C: AF525 cells treated with complete β-elimination protocol. D: AF525 control.
